# Reinvestigation of Reactions of HgPh_2_ with Eu and Yb Metal and the Synthesis of a Europium(II) Bis(tetraphenylborate) †

**DOI:** 10.3390/molecules27217547

**Published:** 2022-11-03

**Authors:** Michal Wiecko, Zhifang Guo, Glen B. Deacon, Peter C. Junk

**Affiliations:** 1 School of Chemistry, Monash University, Clayton, VIC 3800, Australia; 2 College of Science & Engineering, James Cook University, Townsville, QLD 4811, Australia

**Keywords:** europium, ytterbium, lanthanoid organyls, tetraphenylborate anion, mercury organyls

## Abstract

Europium bis(tetraphenylborate) [Eu(thf)_7_][BPh_4_]_2_⋅thf containing a fully solvated [Eu(thf)_7_]^2+^ cation, was synthesized by protolysis of “EuPh_2_” (from Eu and HgPh_2_) with Et_3_NHBPh_4_, and the structure was determined by single-crystal X-ray diffraction. Efforts to characterize the putative “Ph_2_Ln” (Ln = Eu, Yb) reagents led to the synthesis of a mixed-valence complex, [(thf)_3_Yb^II^(*μ*-Ph)_3_Yb^III^(Ph)_2_(thf)]⋅2thf, resulting from the reaction of Yb metal with HgPh_2_ at a low temperature. This mixed-valence Yb^II^/Yb^III^ compound was studied by ^171^Yb-NMR spectroscopy and single-crystal X-ray diffraction, and the oxidation states of the Yb atoms were assigned.

## 1. Introduction

Rare earth metal compounds containing a carbon-to-metal σ-bond have been of considerable interest for many years [1,2,3,4,5,6,7,8,9,10]. The uniquely high reactivity of this mainly ionic bond not only poses a synthetic challenge from the fundamental research viewpoint but also affords an opportunity for widespread synthetic applications [11,12,13,14,15]. Brönstedt and Lewis basic alkyl or aryl ligands can easily be displaced to afford ionic species [9,16,17], offering access to more reactive systems, in particular for the catalysis of olefin polymerization reactions [18,19,20,21,22,23,24]. In this paper, a weakly coordinating tetraphenylborate BPh_4_^-^ anion is used to increase the electropositive character of the central metal and to offer available sites for substrate coordination. In addition, complexes with BPh_4_^−^ offer an interesting reactivity for metalorganic synthesis [25,26,27,28]. In recent years, our group has reported on the synthesis of a number of compounds with BPh_4_^−^ anions [29]. The ionic perfluoroaryllanthanoid(II) tetraphenylborates [Eu(C_6_F_5_)(thf)_6_]BPh_4_ and [Yb(C_6_F_5_)(thf)_5_]BPh_4_ were obtained by treating Yb or Eu metal with HgPh(C_6_F_5_) in the presence of Me_3_NHBPh_4_ [30]. In the course of the present study, we found that the BPh_4_^−^ anion has a stabilizing effect, increasing the thermal robustness of the ionic complexes relative to [Eu(C_6_F_5_)_2_(thf)_5_] and [Yb(C_6_F_5_)_2_(thf)_4_]. Motivated by these results, we were interested in the possible synthesis of analogous Ln^II^ (Ln = Eu, Yb) complexes with an unsubstituted phenyl ligand.

## 2. Results

**Scheme 1:**
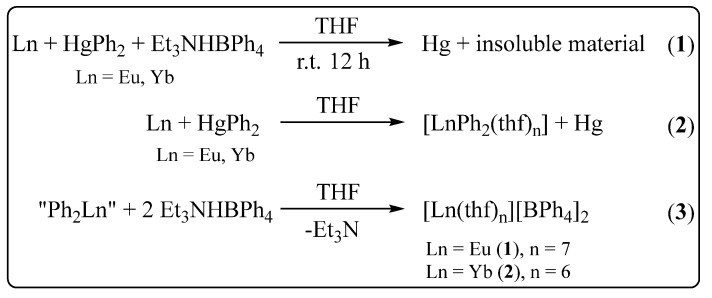
Synthesis of complexes **1**–**2**.

Initially, attempts were made to synthesize the desired complexes, [LnPh(thf)_n_]BPh_4_ by redox transmetallation/ligand exchange reactions of ytterbium or europium metal, HgPh_2_, and Me_3_NHBPh_4_, a synthesis route similar to that used for the preparation of [Eu(C_6_F_5_)(thf)_6_]BPh_4_ and [Yb(C_6_F_5_)(thf)_5_]BPh_4_ [30]. However, along with Hg metal, non-crystalline, poorly soluble products, presumably the bis(tetraphenylborate)s [Ln(thf)_n_][BPh_4_]_2_ (Ln = Eu (**1**), n = 7; Yb (**2**); n = 6), were formed (Scheme 1, Equation (1)). The favoured formation of the Yb bis(tetraphenylborate) [Yb(thf)_6_][BPh_4_]_2_ [31] in various ligand exchange attempts with [Yb(C_6_F_5_)(thf)_5_]BPh_4_ was discussed in [29]. Next, we attempted to synthesize and characterize diphenyl-ytterbium and -europium (Scheme 1, Equation (2)), which could be protolyzed to afford [LnPh(thf)_n_]BPh_4_. According to previous reports, synthesis of [LnPh_2_(thf)_n_] can be achieved by treating Ln metal with HgPh_2_ [32,33]. Following this procedure, the compounds “Ph_2_Yb” and “Ph_2_Eu” were obtained at room temperature as highly pyrophoric black powders. Layering of a THF solution of both compounds with Et_3_NHBPh_4_ resulted in the corresponding bistetraphenylborates as the only isolated product with surprisingly high purity (Scheme 1, Equation (3)). Single crystals of the compounds were collected directly from the reaction mixture, and brown impurities were removed with THF. The ytterbium complex [Yb(thf)_6_][BPh_4_]_2_ **2** was reported in [31], and we also obtained this compound from reactions of [Yb(C_6_F_5_)(thf)_5_]BPh_4_ with protic reagents [29]. The homoleptic complexes **1** and **2** should undergo ligand exchange reactions by displacement of THF. If carried out in non-polar solvents, homoleptic complexes may be targeted.

**Figure 1:**
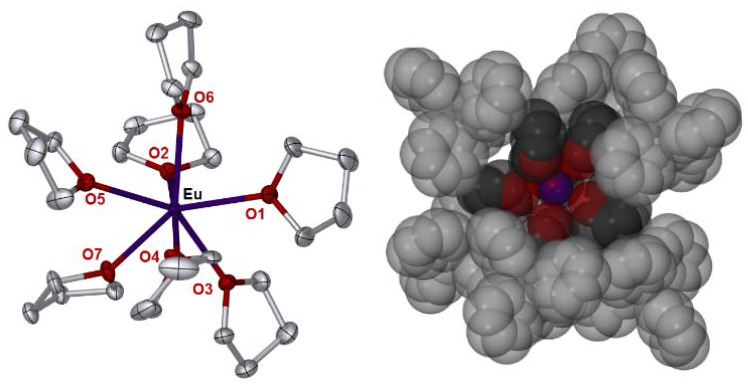
**Left**: Structure of the cation of **1** in the solid state drawn with ellipsoids at 50% probability and hydrogen atoms and anions omitted. **Right**: Space-filling representation of the environment around the [Eu(thf)_7_]^2+^ cation with one BPh_4_^−^ and one THF molecule removed for clarity. Average Eu-O distance: <Eu-O> = 2.57 Å. Selected angles [°]: O1-Eu-O2 106.18(6), O1-Eu-O3 74.84(6), O1-Eu-O4 78.39(6), O1-Eu-O5 131.01(6), O1-Eu-O6 75.14(5), O1-Eu-O7 147.31(6), O2-Eu-O3 85.76(5), O2-Eu-O4 170.19(6), O2-Eu-O5 103.63(5), O2-Eu-O6 78.08(5), O2-Eu-O7 80.49(6), O3-Eu-O4 87.14(6), O3-Eu-O5 145.63(5), O3-Eu-O6 140.13(5), O3-Eu-O7 73.80(6), O4-Eu-O5 78.72(6), O4-Eu-O6 111.66(6), O4-Eu-O7 91.04(6), O5-Eu-O6 74.12(5), O5-Eu-O7 75.29(5), O6-Eu-O7 136.91(6).

A single-crystal X-ray diffraction study of [Eu(thf)_7_][BPh_4_]_2_⋅thf (**1**) (Figure 1, left) showed that it is isostructural to the corresponding Sm^II^ complex reported by Evans [31]. [Eu(thf)_7_][BPh_4_]_2_⋅thf (**1**) crystallized in the monoclinic space group *P*2_1_/*n*. Because the coordination chemistry of rare earth metals is primarily governed by ionic interactions and Sm^II^ and Eu^II^ have virtually the same ionic radius [34], the structures are very similar. The inner coordination sphere of the Eu-centered cation resembles a distorted pentagonal bipyramid. This [Eu(thf)_7_]^2+^ core is surrounded by a total of eight BPh_4_^-^ anions, forming a cube-like shape around the cation (Figure 1 right). The Eu–O distance ranges from 2.5214(17) to 2.6209(16) Å, and the average Eu-O bond length (2.57 Å) in **1** is comparable to that of [Sm(thf)_7_][BPh_4_]_2_⋅thf [31], owing to the similar sizes of Sm^2+^ and Eu^2+^ [34].

**Scheme 2:**
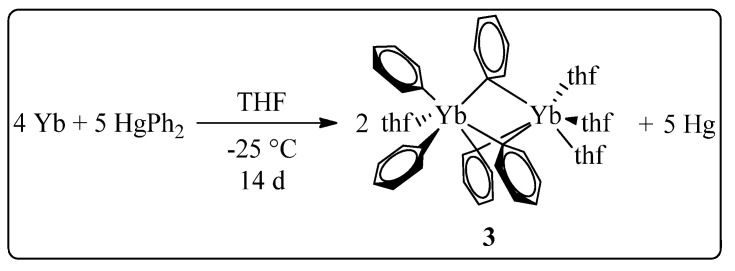
Synthesis of complex **3**.

We were unable to directly characterize the putative “[LnPh_2_(thf)_x_]” products from the redox transmetallation reaction (Scheme 1, Equation (2)). Considering the temperature sensitivity of lanthanoid aryls and alkyls and the possibility of ether cleavage reactions [35,36,37,38], the redox transmetallation reaction was repeated at −25 °C. At this temperature, a reaction of HgPh_2_ with Eu or Yb was only observed at a concentration of at least ~0.5 mol/L. Storage of this slurry mixture at −25 °C for two weeks under daily sonication at r.t. for ~1 min. resulted in the formation of large red crystals of the mixed valent species [Yb_2_Ph_5_(thf)_4_]⋅2thf (**3**) (Scheme 2), a complex previously isolated by Bochkarev et al. without THF of crystallization, after synthesis by a reaction of ytterbium naphthalenide with HgPh_2_ [39]. Our new facile synthesis route offers a unique opportunity for future selective syntheses of +2/+3 mixed-valence Yb compounds by protolytic ligand exchange reactions of [Yb_2_Ph_5_(thf)_4_]. No Eu compound similar to **3** or any solely divalent species was isolated from an analogous synthesis.

**Figure 2:**
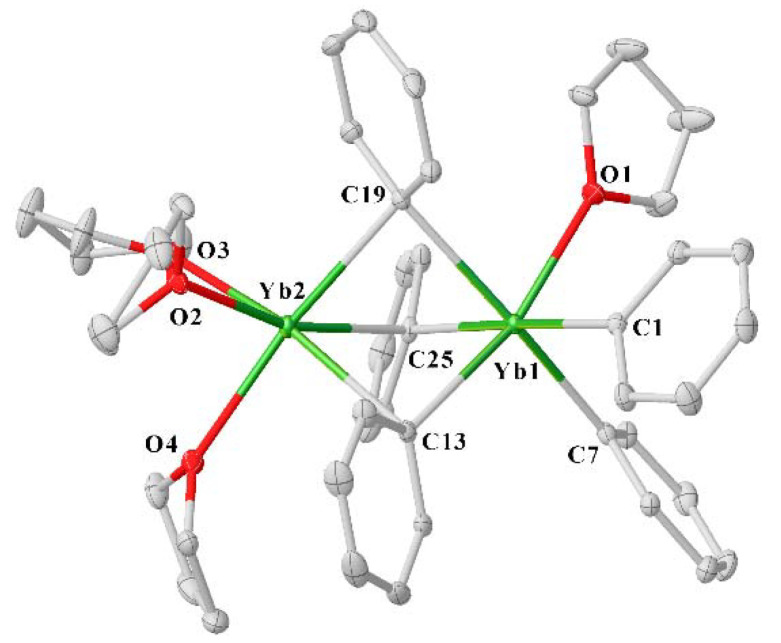
X-ray crystal structure of compound **3**. Thermal ellipsoids are shown at 40%. Hydrogen atoms have been removed for clarity.

Compound **3** crystallized in the monoclinic space group *C*2/*c* with one whole molecule in the asymmetric unit (Figure 2), whereas the structure reported by Bochkarev was solved and refined in monoclinic space group *P*2_1_ with two whole molecules in the asymmetric unit (one had two disordered THF ligands) [39]. In the structure, two six-coordinate Yb atoms are bridged by three phenyl ligands, each through a single C atom. Yb1 has two terminal phenyl ligands and one THF donor, whereas Yb2 has only three terminal THF ligands. The simplest explanation for the stoichiometry is that one Yb atom is Yb^II^ and the other is Yb^III^, and these should be distinguishable based on the ionic radius difference of 0.15 Å between six-coordinate Yb^2+^ and Yb^3+^ [34]. However, the reality is more complex. Relevant bond lengths are listed in Table 1, together with comparable data reported by Bochkarev for molecule Yb3,4 of [Yb_2_Ph_5_(thf)_4_] [39]. Both Yb1 and Yb2 have terminal THF ligands, where Yb1-O1 is 2.400(6) Å, whereas <Yb-O2,3,4> is 2.45 Å, which is a difference much less than that expected if Yb1 is trivalent and Yb2 is divalent. The corresponding difference for the reported complex is much larger, at 0.14 Å [39], but the difference is not significant according to the 3 e.s.d. criterion. On the other hand, the terminal Yb1-C1,7 bond lengths of **3** (2.446(8), 2.445(7)) correspond closely to those of the six-coordinate anionic Yb^III^ complex [YbPh_4_(dme)]^−^ (2.427(9)-2.475(9) Å) [40]. They are also close to the Yb-C bond lengths (2.398(5)–2.423(5) Å) of six-coordinate [Yb^III^Ph_3_(thf)_3_] [40,41]. In the divalent six-coordinate complex [Yb(C_6_F_5_)_2_(thf)_4_], Yb-C is considerably longer, at 2.649(3) Å [42]. Accordingly, the Yb-C bond lengths provide evidence that Yb1 is +III; therefore, Yb2 is in the +II oxidation state. The reported data support the view that Yb3 (Table 1) is trivalent, despite the high e.s.d. values [39]. The small difference in the terminal Yb-O bond lengths despite the differing oxidation states proposed above is consistent with the reported data; Yb-O is only marginally shorter in [YbPh_3_(thf)_3_] (2.381(3)-2.413(3) Å) [43] than in [Yb(C_6_F_5_)_2_(thf)_4_] (2.428(2)-2.440(2) Å) [30].

**Figure 3:**
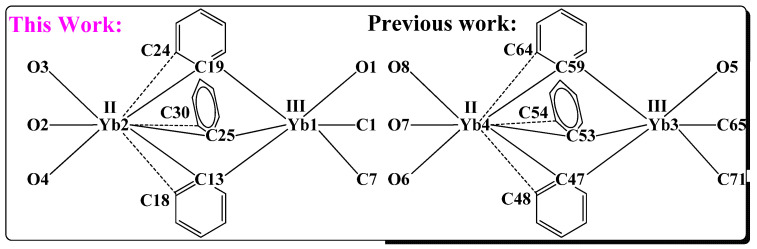
Comparison of the numbering of complex **3** and [Yb_2_Ph_5_(thf)_4_] [39].

In the case of the bridging phenyl groups, C13, 19, and 25 are bound more closely to Yb1 than Yb2, by 0.176, 0.068, and 0.019 Å, respectively (Table 1, Figure 3), the last difference not being significant; however, overall, these data are consistent with the above oxidation state assignments for Yb1 and Yb2. In addition an *ortho* carbon (C18, C24, and C30), of the phenyl rings of C13, 19, and 25 approach Yb2 at 2.993(4)-3.130 Å (Table 1), whereas the other *ortho* carbon (C14, 20, and 26) of the same rings is more distant from Yb1, despite the higher oxidation state of Yb1. The Yb2-*ortho*-C contacts are in the range for intramolecular Yb^II^-π-C(Ph) interactions [44,45,46,47,48,49], whereas the Yb1-C distances are too long for credible Yb^III^-C π interactions [44]. There are also possible agostic interactions with the *ortho* C-H groups on the phenyl rings of C(18,24,30), as the Yb(2)-H distances (Table 1) are just within the sum of the van der Waals radius of hydrogen (1.20 Å) [50] and the metallic radius (pseudo-van der Waals radius) of ytterbium (1.94 Å) [51]. The three bridging phenyl groups lie outside of the plane generated by C(13), C(19), and C(25) by 10, 17, and 9°, respectively, and all twist in the same direction towards Yb(2) in a propellor-like fashion. In summary, π and possible C-H-Yb agostic interactions reinforce the bridging C-Yb2 interaction, leading to a closer approach of the bridging arene rings to divalent Yb2. If the C13 Ph group, which has, by far the greatest difference between the Yb1-C and Yb2-C bond lengths, is allocated to Yb1, then the structure would be an association of [YbPh_3_(thf)] and [YbPh_2_(thf)_3_], an outcome consistent with the proposal of Bochkarev that the complex is formed by an association of YbPh_3_ and YbPh_2_ [39]. Applying the 3 e.s.d. criterion to the reported [Yb_2_Ph_5_(thf)_4_] structure, the separations of Yb3 and Yb4 from the same bridging C atom [39] are essentially indistinguishable. In the present structural refinement, the average Yb2-C bond distance is 2.64 Å (2.66 Å for Bochkarev’s structure [39]), whereas the analogous Yb1-C average is 2.51 Å (2.48 Å for that reported in [39]), and the average Yb-O distances are 2.45 and 2.400(6) Å for Yb2 and Yb1, respectively (2.44 and 2.30 Å for those reported in [39]).

**Figure 4:**
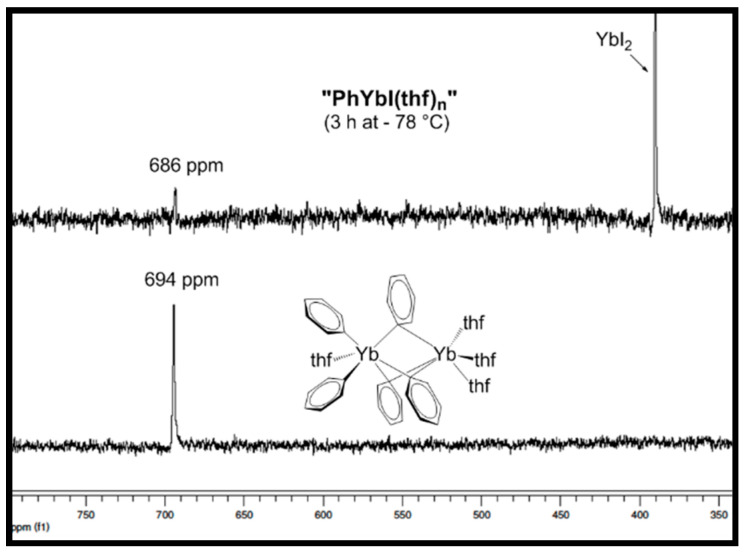
^171^Yb-NMR spectra of “PhYbI(thf)_n_” (top, −78 °C) and complex **3** (bottom, −30 °C).

**Scheme 3:**
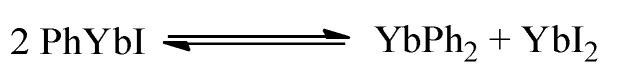
Schlenk equilibrium in the PhYbI system (coordinated solvent not shown) [41].

The ^171^Yb NMR spectrum suggests that complex **3** dissociates into two neutral species of differing oxidation states, namely YbPh_2_ and YbPh_3_. As paramagnetic Yb^III^ complexes have not been observed in ^171^Yb NMR spectra, it is unlikely that a mixed-valence Yb_2_Ph_5_ species would be observed, owing to bridging of Yb^II^ through phenyl groups to Yb^III^. In the ^171^Yb NMR spectrum of the compound (Figure 4), a single resonance is observed at δ = 694 ppm. This value is similar to a resonance (686 ppm) in the ^171^Yb NMR spectrum of the reaction mixture from Yb metal and iodobenzene in THF at -78°C, a system that reacts as “PhYbI(thf)_n_” [40]. Because the spectrum also has a resonance at 388 ppm attributable to YbI_2_ [40,41,52], the resonance at 686 ppm can be assigned to [YbPh_2_(thf)_n_], the product with YbI_2_ of the Schlenk equilibrium (Scheme 3). This, in turn, leads to the assignment of 694 ppm of **3** to [YbPh_2_(thf)_n_], with coproduct YbPh_3_ silent in the ^171^Yb NMR spectrum, owing to paramagnetism. The chemical shift lies between those of four-coordinate [Yb(2,6-Ph_2_C_6_H_3_)_2_(thf)_2_] reported by Niemeyer (927 ppm) [52], and that of six-coordinate [Yb(C_6_F_5_)_2_(thf)_4_] [ (463 ppm) [30,42]. Because coordination number is a key determinant of the chemical shift in the ^171^Yb NMR spectra within a series of related compounds [53,54] and [YbPh_2_(thf)_n_] can be expected to have a coordination number more similar to that of [Yb(C_6_F_5_)_2_(thf)_4_] than the sterically crowded [Yb(2,6-Ph_2_C_6_H_3_)_2_(thf)_2_], the observed value is probably reasonable for n = 3 (5 coordination), given the differing electron-withdrawing properties of C_6_F_5_ and Ph, or even n = 4.

## 3. Materials and Methods

### 3.1. General Considerations

All manipulations were performed with the rigorous exclusion of oxygen and moisture in flame-dried glassware with greaseless stopcocks, either on a dual-manifold Schlenk line or in an argon-filled Saffron glovebox. All solvents were predried over Na wire, stored over Na/K benzophenone ketyl, and distilled prior to use. “Ph_2_Ln” (Ln = Eu, Yb) and “PhYbI(thf)_n_” were prepared according to the procedure described in [32,33,40,41]. NMR spectra were obtained using a *Bruker DPX* 300 MHz spectrometer. ^171^Yb-NMR spectra are referenced to 0.15 M [Yb(C_5_Me_5_)_2_(thf)] in THF/10 % C_6_D_6_ [53].

### 3.2. Synthesis of [Eu(thf)_7_][BPh_4_]_2_⋅thf (1)

“Ph_2_Eu” (60 mg) was dissolved in THF (3 mL) in a glass tube. The solution was slowly topped with a solution of Et_3_NHBPh_4_ (100 mg, 0.24 mmol) in 7 mL of THF. The solutions were allowed to mix over a period of 3 days. The remaining dark solution was decanted, evaporated and the resulting off-white crystalline solid was washed with 2 mL of THF, affording 71 mg (55 % based on Et_3_NHBPh_4_) of compound **1**. Single crystals suitable for X-ray diffraction were found in this solid. IR (Nujol, cm**^−^**^1^): 1577 (m), 1542 (m), 1307 (w), 1261 (s), 1156 (s), 1069 (w), 1030 (w), 856 (w), 800 (w), 747 (s), 711 (s). Anal. Calcd. for C_76_H_96_B_2_EuO_7_ (1295.66 g/mol, loss of THF of crystallization): C 70.48, H 7.47; found: C 70.85, H 7.68. 

### 3.3. Synthesis of [Yb(thf)_6_][BPh_4_]_2_ (2)

“Ph_2_Yb” (60 mg) was dissolved in THF (3 mL) in a glass tube. The solution was slowly topped with a solution of Et_3_NHBPh_4_ (100 mg, 0.24 mmol) in 7 mL of THF. The solutions were allowed to mix over a period of 3 days. The remaining dark solution was decanted, evaporated and the resulting yellow crystalline solid was washed with 2 mL of THF, affording compound **2**. Single crystals suitable for X-ray diffraction were found in this solid, and 2 was identified by unit cell [31].

### 3.4. Synthesis of [Ph_5_Yb_2_(thf)_4_]⋅2thf (3)

Ytterbium metal (260 mg, 1.5 mmol) and HgPh_2_ (355 mg, 1.0 mmol) were suspended in 2 mL of THF. Upon sonication at r.t. for 2 h, the mixture developed a red color and was thereafter cooled to −25 °C. Daily sonication for 1 min at r.t. and storage at −25 °C resulted in the formation of mercury metal and a dark red crystalline solid. The remaining solution was decanted, and the residue was treated with 10 mL of THF and decanted from the mercury metal. Concentration of the solution to 5 mL resulted in the formation of large red crystals of 3∙(thf)_2_ suitable for X-ray diffraction. Isolated yield: 209 mg (41 %). ^171^Yb-NMR (thf, 52.6 MHz, −30 °C): δ = 694 ppm. 

### 3.5. X-ray Crystallography

Complexes **1** and **3** were measured on a Bruker APEX-II CCD diffractometer equipped with graphite-monochromated Mo-Kα radiation (λ = 0.71073 Å) at 123 K and mounted on a fiber loop in crystallography oil. Absorption corrections were completed using the Apex II program suite with SADABS [55]. Structural solutions were obtained by SHELXT [56] using full-matrix least-squares methods against F2 with SHELX2018 [56] in conjunction with the Olex2 [57] graphical user interface. All hydrogen atoms were placed in calculated positions according to the riding model. 

Crystal data for **1** and **3**: 

**1**: C_80_H_104_B_2_EuO_8_, *M* = 1367.21, yellow block, 0.5 × 0.4 × 0.2 mm^3^, monoclinic, space group *P*2_1_/*n* (No. 14), *a* = 18.2175(6) Å, *b* = 19.1237(6) Å, *c* = 20.2661(6) Å, *β* = 94.6320(10)°, *V* = 7037.4(4) Å^3^, *Z* = 4, *ρ*_c_ = 1.290 g/cm^3^, *μ* = 0.947 mm**^−^**^1^, *F*_000_ = 2884, Bruker X8 Apex II CCD, MoK*_α_* radiation, λ = 0.71073 Å, *T* = 123(1)K, 2*θ*_max_ = 52.0°, 202,209 reflections collected, 13,450 unique (R_int_ = 0.0622), Final *GooF* = 1.035, *R_1_* = 0.0323 [I > 2σ(I)], *wR_2_* = 0.0681.

**3**: C_54_H_73_O_6_Yb_2_, *M* = 1164.20, dark red block, 0.50 × 0.30 × 0.30 mm^3^, monoclinic, space group *C*2/*c* (No. 15), *a* = 40.713(8) Å, *b* = 11.271(2) Å, *c* = 22.724(5) Å, *β* = 104.70(3)°, *V* = 10,086(4) Å^3^, *Z* = 8, *ρ*_c_ = 1.533 g/cm^3^, *μ* = 3.733 mm**^−^**^1^, *F*_000_ = 4680, Bruker X8 Apex II CCD, MoK*_α_* radiation, λ = 0.71073 Å, *T* = 123(1) K, 2*θ*_max_ = 55.8°, 39,023 reflections collected, 11,781 unique (R_int_ = 0.0324), Final *GooF* = 1.006, *R_1_* = 0.0282 [I > 2σ(I)], *wR_2_* = 0.0590.

## 4. Conclusions

In conclusion, we prepared the ionic Eu(II) complex [Eu(thf)_7_][BPh_4_]_2_⋅thf. In an attempt to characterize the previously described “Ph_2_Ln” compounds, which can be used to prepare [Ln(thf)_n_][BPh_4_]_2_ (Ln = Eu, Yb), an improved synthesis of the Yb^II^/Yb^III^ compound [Yb_2_Ph_5_(thf)_4_] was developed. The Eu complex **1** (and the Yb complex **2**) should be a source of homoleptic complexes via displacement of THF by neutral ligands in non-polar solvents, whereas **3** may yield other mixed-oxidation-state Yb complexes via protolysis reactions, whereby the phenyl groups of **3** are replaced by ligands (L) (e.g., L = OAr, OR, pyrazolate, amidinate, etc.) by reaction with the corresponding proligand (HL).

## Figures and Tables

**Table 1:** Selected bond lengths of complex **3** and the reported structure of [Yb_2_Ph_5_(thf)_4_].

This Work		Ref [39] *	
Bond	Length (Å)	Bond	Length (Å)
Yb1-C1	2.446 (8)	Yb3-C65	2.39 (4)
Yb1-C7	2.445 (7)	Yb3-C71	2.46 (3)
Yb1-O1	2.400 (6)	Yb3-O5	2.30 (2)
Yb1-C13	2.489 (9)	Yb3-C59	2.48 (3)
Yb1-C19	2.573 (8)	Yb3-C53	2.54 (3)
Yb1-C25	2.583 (8)	Yb3-C47	2.51 (4)
Yb2-C13	2.665 (8)	Yb4-C59	2.68 (3)
Yb2-C19	2.641 (10)	Yb4-C53	2.55 (3)
Yb2-C25	2.602 (8)	Yb4-C47	2.75 (4)
Yb1-O4	2.452 (6)	Yb4-O6	2.44 (2)
Yb2-O2	2.434 (6)	Yb4-O7	2.38 (3)
Yb2-O3	2.462 (6)	Yb4-O8	2.50 (3)
Yb2^…^C18	3.034 (4)	Yb4^…^C48	3.08 (4)
Yb2^…^C24	2.995 (4)	Yb4^…^C64	2.93 (3)
Yb2^…^C30	3.130 (4)	Yb4^…^C54	3.25 (4)
Yb1-C14 ^a^	3.350 (4)	Yb3-C52 ^a^	3.45 (4)
Yb1-C20 ^a^	3.244 (4)	Yb3-C60 ^a^	3.16 (4)
Yb1-C26 ^a^	3.412 (4)	Yb3-C58 ^a^	3.39 (5)
Yb2^…^H24	2.785		
Yb2^…^H18	2.921		
Yb2^…^H30	3.060		

* There are two similar molecules of [Yb_2_Ph_5_(thf)_4_] in the reported structure. As some THF ligands were disordered in the molecules labelled Yb1 and Yb2, we only made a comparisonwith the non-disordered molecule Yb3 and Yb4. ^a^ Nonbonding.

## Data Availability

Crystal data can be obtained free of charge from the Cambridge Crystallographic Data Centre (CCDC 2211517 for 1 and 2211518 for 3).
